# ZnO@SnO_2_ engineered composite photoanodes for dye sensitized solar cells

**DOI:** 10.1038/srep14523

**Published:** 2015-09-30

**Authors:** R. Milan, G. S. Selopal, M. Epifani, M. M. Natile, G. Sberveglieri, A. Vomiero, I. Concina

**Affiliations:** 1 Department of Information Engineering, University of Brescia - via Valotti 9, 25133 Brescia, Italy; 2 CNR-INO SENSOR Laboratory, via Branze 45, 25131 Brescia Italy; 3 Istituto per la Microelettronica e Microsistemi, IMM-CNR, via Monteroni, 73100 Lecce, Italy; 4 CNR-IENI, Department of Chemical Sciences, University of Padova, via F. Marzolo 1, 35131 Padova, Italy; 5 Department of Engineering Science and Mathematics, Luleå University of Technology, 97187 Luleå, Sweden

## Abstract

Layered multi-oxide concept was applied for fabrication of photoanodes for dye-sensitized solar cells based on ZnO and SnO_2_, capitalizing on the beneficial properties of each oxide. The effect of different combinations of ZnO@SnO_2_ layers was investigated, aimed at exploiting the high carrier mobility provided by the ZnO and the higher stability under UV irradiation pledged by SnO_2_. Bi-oxide photoanodes performed much better in terms of photoconversion efficiency (*PCE*) (4.96%) compared to bare SnO_2_ (1.20%) and ZnO (1.03%). Synergistic cooperation is effective for both open circuit voltage and photocurrent density: enhanced values were indeed recorded for the layered photoanode as compared with bare oxides (*V*_*oc*_ enhanced from 0.39 V in case of bare SnO_2_ to 0.60 V and *J*_*sc*_ improved from 2.58 mA/cm^2^ pertaining to single ZnO to 14.8 mA/cm^2^). Improved functional performances of the layered network were ascribable to the optimization of both high chemical capacitance (provided by the SnO_2_) and low recombination resistance (guaranteed by ZnO) and inhibition of back electron transfer from the SnO_2_ conduction band to the oxidized species of the electrolyte. Compared with previously reported results, this study testifies how a simple electrode design is powerful in enhancing the functional performances of the final device.

Dye sensitized solar cells (DSSCs) have raised remarkable interest since 1991, after the publication of the pioneering study by O’Regan and Gratzel^1^. These photoelectrochemical cells promise to be effective alternative to silicon-based photovoltaics (PV), thanks to their low cost (as for both applied materials and fabrication processes) and to their reduced environmental impact^2^,^3^,^4^. Although their performances are lower (photoconversion efficiency not overcoming 14%) than traditional PV^5^, due to intrinsic limitation in charge transport, efforts devoted to improving the devices have not been reduced.

The most studied system exploits a thick film (12–18 μm) of TiO_2_ nanoparticles (NPs) as photoanode, but a certain interest is being focused also on other potentially suitable semiconducting metal oxides such as ZnO, SnO_2_, Nb_2_O_5_ and WO_3_ 6,7,8,9,10,11. Among them, ZnO and SnO_2_ are the most appealing candidates, due to their higher electron mobility, as compared with TiO_2_^12^,^13^ and specific advantages, such as a ZnO band gap and band positioning energetically similar to TiO_2_ 14, and a larger band gap (3.8 eV *vs* 3.2 eV) of SnO_2_ as compared with TiO_2_, which should guarantee higher stability under UV illumination^15^. However, until now, device performances recorded by applying these oxides have been lower than those provided by TiO_2_ NPs: these results come from different issues related to ZnO and SnO_2_. In ZnO one reason adduced by several authors is the instability of this material in acidic media, i.e prolonged immersion of ZnO in N719 dye leads to the formation of a Zn^2+^-N719 complex layer over ZnO surface that affects the electron injection rate^16^, although a major role in reduced performances seems played by injection itself^17^,^18^.

In the case of SnO_2_, unsatisfying performances are related to recombination processes and unfavorable band alignment of the oxide with respect to the lowest unoccupied molecular orbital (LUMO) of the Ru-based dye N719, which still is the most widely applied dye in DSSCs, resulting in quite reduced photovoltages^19^.

A potentially powerful strategy to overcome the single limitations of ZnO and SnO_2_ is the application of both materials simultaneously. This approach has gained fame since the study by Tennakone and coworkers appeared in 1999^20^, in which Authors successfully applied as photoanode a porous film composed of mixed ZnO-SnO_2_, featuring a remarkably high photoconversion efficiency (8%). This result still retains all its value, being the highest performance ever recorded for a SnO_2_-based DSSC. However, despite several attempts, up to now nobody had been able to reproduce this remarkable result. Grätzel’s group studied the surface modification of nanocrystalline SnO_2_ with a thin layer of different metal oxides, concluding that major improvement of this configuration can be ascribed to enhanced dye uptake (hence generated photocurrent) together with the suppression of charge recombination from SnO_2_ to iodine-based electrolyte^21^. Similar approach was proposed by Zaban and coworkers^22^, who exploited presumably core@shell M_x_O_y_@SnO_2_ nanoparticles looking for privileged path for photogenerated charge collection. Basic concept exploited in these mixed metal oxide systems is the creation of paths featuring energy as low as possible for electrons diffusing through the photoanodes, by inducing more favorable band alignment within the whole system (dye/metal oxide 1/metal oxide 2). This is also the main reason behind the usually adopted practice to deposit a layer of TiO_2_ atop SnO_2_ anode through a TiCl_4_ treatment.

More recently, a strengthened interest is being paid to SnO_2_-based DSSCs and different shape/configurations have been tested, among which SnO_2_ hierarchical octahedra (featuring different performances according to structure sizes)^23^, SnO_2_ flowers and fibers^24^, SnO_2_ nanorods^25^.

Previous literature in the field demonstrates, as mentioned above, the overall benefit induced by the presence of ZnO, but without investigating whether different amounts of this material induce a systematic effect on device functional performances.

Herein, we demonstrate how a quite simple approach may drastically enhance cell performances in ZnO@SnO_2_ DSSCs, without the need to resort to either complex synthetic approaches or core@shell systems. An elementary wet chemical synthesis was indeed exploited to generate a first SnO_2_ NPs layer, extremely homogeneous as for both sizes and shape of NPs, while commercial ZnO polydispersed microparticles were applied as effective capping layer to both reduce the back electron recombination from SnO_2_ and enhance electron injection from the dye. Keeping fixed the overall photoanode thickness, we modulated the amounts of SnO_2_ and ZnO separately, in a systematic investigation to optimize cell performances. Electrochemical impedance analysis strongly suggests that benefits are not coming from electronic band engineering, as previously suggested by other studies, but simply from the enhancement of SnO_2_ chemical capacitance provided by ZnO addition, which however results in lowering the recombination resistance of this latter oxide.

## Results and Discussion

**Figure 1:**
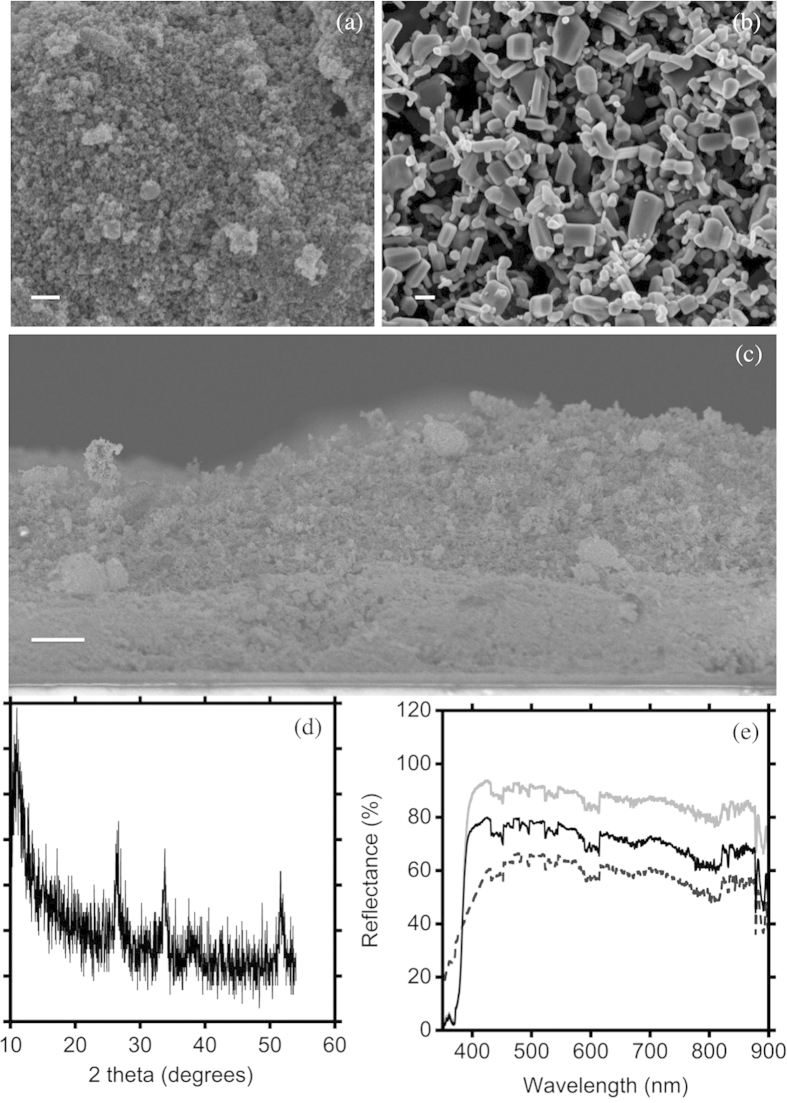
SEM micrographs of (**a**) SnO_2_ nanoparticles; (**b**) ZnO commercial particles. (**c**) Cross sectional view of a bi-oxide photoanode. Scale bars: (**a**) and (**b**) 200 nm; (**c**) 5 μm. (d) XRD pattern of SnO_2_ nanoparticles. (**e**) Reflectance spectra of pure ZnO (grey line), pure SnO_2_ (dashed black line) and ZnO@SnO_2_ (solid black line) photoanodes.

Figure 1 shows the SEM analysis of the metal oxide structures applied as photoanodes in this work. SnO_2_ NPs constitute a compact network after annealing characterized by homogenous particles (Fig. 1a). The GIXRD patter (Fig. 1d) reveals a good crystallinity of SnO_2_ NPs with crystallite size of 21 nm in accordance with SEM evaluation. On the other hand, commercially available ZnO structures are applied as capping layer, which are polydispersed in both sizes and shapes (Fig. 1b, aggregate size in the range 20 to 500 nm), thus acting as scattering centers for light^26^. Cross section SEM analysis clearly shows the bi-layered composition of the proposed photoanode architecture: SnO_2_ forms a quite compact scaffold, on top of which a more porous layer of ZnO is lying.

Photoanode structure affects the incident light reflection (Fig. 1e): ZnO microparticles show an extremely high reflectance (83–90%) in all the UV-Vis range, thanks to the high light scattering from particles in the same range of the incident radiation, while the smaller SnO_2_ nanoparticles (with lateral dimensions far below light wavelength) exhibit much lower values (less than 60%) in the same broad range. Bi-oxide photoanode behavior is in between the two oxides, as expected, due to the presence of a weighted fraction of film (ZnO) inducing high scattering, and the other part (SnO_2_) being less effective on this: the layered structure is thus able to average the behavior towards light capture of ZnO and SnO_2_, somehow defining an equilibrium as for light reflectance between the components.

**Figure 2:**
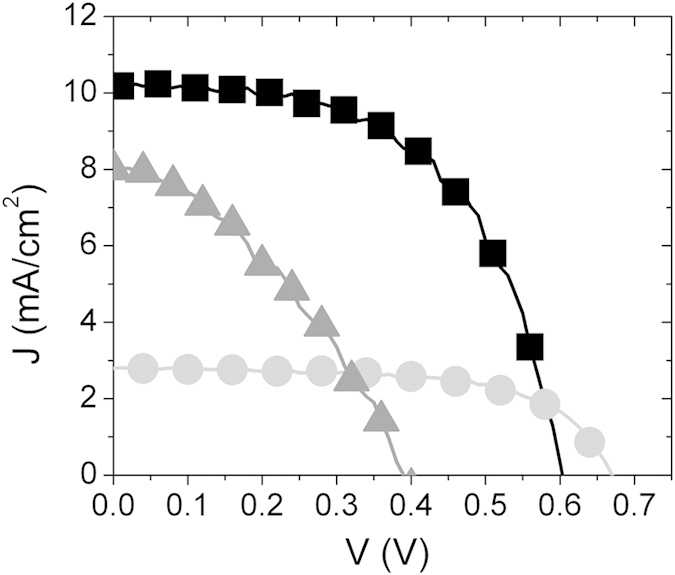
J–V curves of DSSCs based on ZnO (grey line), SnO_2_ (light grey line) and a mixed ZnO@SnO_2_ network composed of 3 ZnO and 3 SnO_2_ layers (3@3 sample, black line). All the photoanodes were sensitized for 6 h.

Devices exploiting single and layered oxides as photoanodes were tested, in order to explore the corresponding functional parameters (Fig. 2 and Table 1). Different bi-oxide photoanode compositions were then considered, in which the overall thickness was kept constant (~20 μm) during sample preparation, while changing the relative amount of ZnO and SnO_2_. Specifically, the following samples were considered: 0@6, 1@5, 2@4, 3@3, 6@0, where the numbers stand for the number of layers tape cast on the conducting glass.

We carried out first a comparison between pure SnO_2_, pure ZnO and a sample composed of the same number of ZnO and SnO_2_ layers (i.e. samples 0@6, 6@0, 3@3). The maximum open circuit photovoltage (*V*_*oc*_) in SnO_2_ samples was rather low (0.39 V) compared to pure ZnO (0.67 V). On the contrary, a good photocurrent density was recorded for the pure SnO_2_ DSSC (as high as 8.00 mA/cm^2^), compared to ZnO (2.58 mA/cm^2^). The trend in *V*_oc_ can be understood in terms of the position of the conduction band (CB) of the ZnO and SnO_2_, respectively^17^. The low *J*_sc_ value recorded for the pure ZnO photoanode is probably ascribable to the polydispersity of the applied microparticle structure, which results, as visible in SEM analysis (Fig. 1b), in an overall open architecture, which does not favor charge transport, generating improper electron paths. Polydispersion in sizes has been demonstrated^27^,^28^ to be extremely beneficial in ZnO-based DSSCs because it increases light capture and charge photogeneration, while a compact network is critical for electron transport, otherwise photogenerated charges tend to recombine during their transport to the electrodes.

Application of a composite bi-oxide layered photoanode results in dramatic improvement of the overall device performances (Fig. 2 and Table 1). In particular, *V*_oc_ was 0.60 V, *J*_sc_ was 10.28 mA/cm^2^ and FF was 57%. The PCE was 3.53%, i.e. about three times larger than for pure SnO_2_ and three and a half times than pure ZnO. These results can be tentatively explained by considering the optimal position of ZnO conduction band, with respect to N719 LUMO, which guarantees for *V*_*oc*_ enhancement, compared to pure SnO_2_. Increased photocurrent calls for good electron transport, guaranteed by the tightly connected SnO_2_ network.

**Figure 3:**
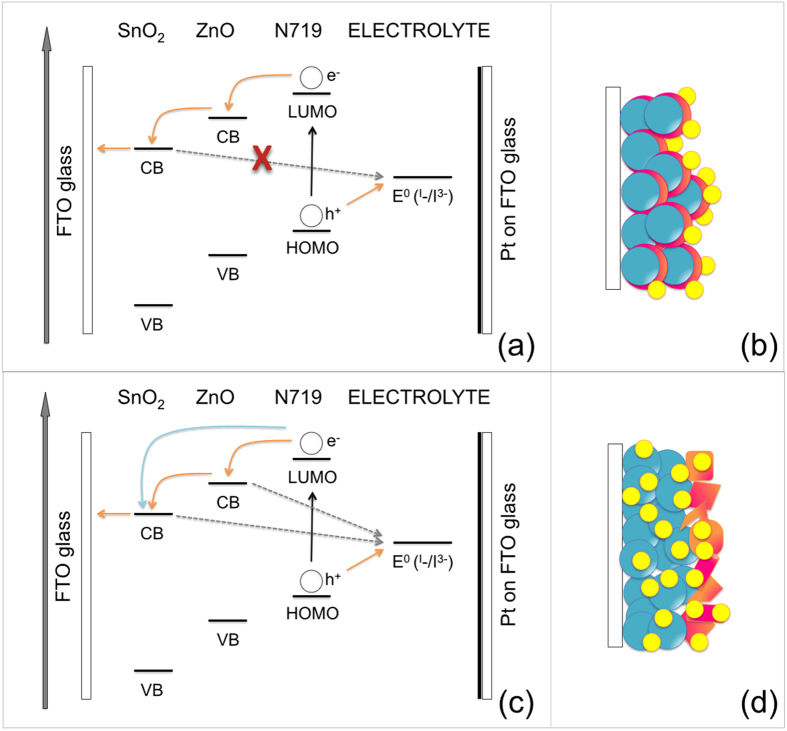
Proposed band energy scheme and main charge transport processes for (**a**) hemi core-shell ZnO-SnO_2_ structures and (**c**) layered architecture proposed in this work. (**b**) and (**d**) show the two configurations theoretically corresponding to band energy diagrams reported in (**a**) and (**c**), respectively (blue spheres: SnO_2_; orange structures: ZnO; yellow spheres: dye N719).

It should be indeed pointed out that several Authors claimed for an overall band alignment effect induced by the addition of ZnO to SnO_2_ in photoanode composition. This hypothesis deserves to be discussed, since it has been broadly debated in previous literature on the topic. As mentioned above, the first work exploring the properties of mixed ZnO/SnO_2_ photoanodes^20^ hypothesized that an effect of band gap engineering might be induced by surrounding the SnO_2_ particles with ZnO species, favoring the charge injection from the N719 LUMO to the ZnO CB and then transferring the photogenerated electrons to the SnO_2_ CB. This favorable band alignment would result in two relevant improvements, as schematically illustrated in Fig. 3a: the first advantage would be the possibility to properly inject photogenerated electrons from N719 to SnO_2_ through the ZnO (which however still presents issues as for injection in itself) and the second relevant advantage would be the elimination of the so-called back recombination between SnO_2_ CB and the electrolyte redox couple (represented by the dashed grey arrow in Fig. 3a), as the outer ZnO shell acts as effective tunneling barrier between the SnO_2_ NP and the electrolyte.

However, this schematic would be realistic only for very particular ZnO/SnO_2_ architectures, as those represented in Fig. 3b, in which SnO_2_ is partially surrounded by ZnO and dye is exclusively adsorbed on ZnO. Additional constrain would be the contact between SnO_2_ and FTO, where ZnO should not be involved at all, in order to avoid an energy gap between SnO_2_ CB and ZnO CB, as it is for instance in core-shell SnO_2_-ZnO systems, through which electron is not allowed to run. In photoanode structures different from those above described, such for instance disordered SnO_2_-ZnO networks or the layered configuration herein proposed (Fig. 3d), there are several limitation as for charge transport processes that should be taken into account. In those cases, dye would be anchored to both metals and then electron injection would take place in both SnO_2_ and ZnO, as well as back recombination with electrolyte (as depicted in Fig. 3c).

**Figure 4:**
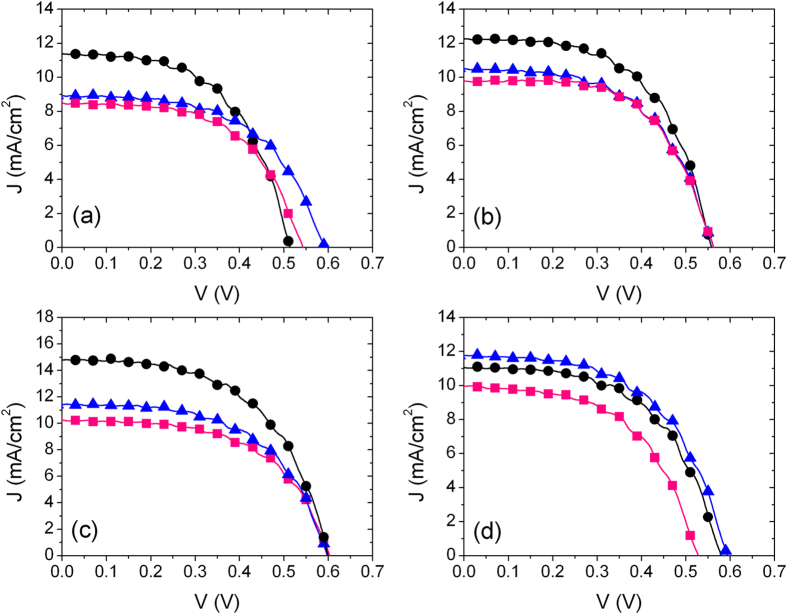
*J–V* characteristics of layered ZnO@SnO_2_ devices (1@5: black circles; 2@4: blue triangles; 3@3: pink squares) dye loaded for different times: (**a**) 2 h, (**b**) 4 h, (**c**) 6 h, (**d**) 10 h.

Based on the preliminary result reported in Fig. 2, supporting the beneficial role of ZnO even as simple “capping” layer for SnO_2_ based photoanodes, we performed a systematic study on the possible effect exerted by the layered oxide configuration by changing the relative number of ZnO@SnO_2_ layers and modulating the time applied for dye uptake. Device functional parameters, as well as applied sensitization times are reported in Table 2 and Fig. 4.

Sensitization time could indeed be critical in ZnO@SnO_2_ cells: differently from TiO_2_-based DSSCs, best performing device based on ZnO has been sensitized for a very reduced time (2 h)^28^, due to the instability of ZnO in the acidic environment determined by the dye solution applied for sensitization. SnO_2_ can instead be safely exposed for longer times to the dye solution without damage of the metal oxide scaffold^21^. In the present work, the effect of dye loading time on device performances was evaluated, since a compromise had to be found between the needs of preserving the ZnO capping layer, on one hand, and reaching a good dye uptake for SnO_2_, on the other.

**Figure 5:**
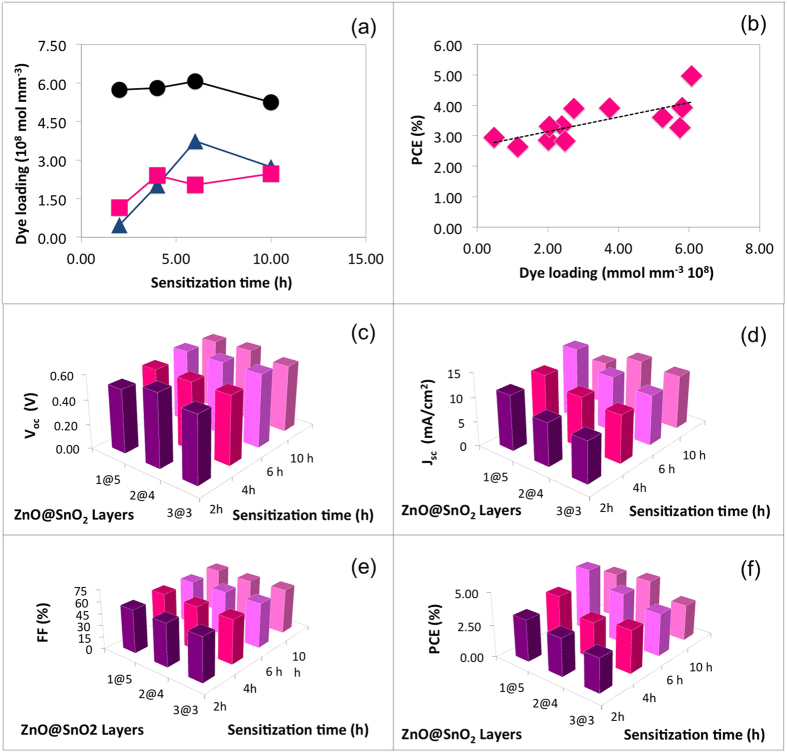
(**a**) Effect of configuration of layered structure of ZnO@SnO_2_ network on dye loading as a function sensitization time (black circles: 1@5, pink squares: 1@4 and blue upper triangles: 3@3). (**b**) PCE *vs* dye loading based on data in Table 2, merged in a single graph, irrespective of the structure of the photoanode. The solid line is the linear fitting of the experimental data. (**c**) to (**f**) Functional parameters of the DSSCs as a function of their structure and sensitization time: *V*_oc_ (**c**); *J*_sc_ (**d**); FF (**e**); PCE (**f**).

Three different layered architectures of ZnO@SnO_2_ (1@5, 2@4, 3@3) were thus sensitized for 2 h, 4 h, 6 h and 10 h. All the tested devices, irrespective of the photoanode architecture, showed best functional performances when sensitized for 6 h (see Table 2). One of the motivations behind this result is the maximized dye loading after 6 h sensitization, as clearly shown in Fig. 5b, in which PCE is reported as a function of dye loading, merging data coming from different photoanode structure and sensitization time. It is quite worth noting that PCE linearly increases with the increased dye uptake, and data from different photoanodes and different sensitization time are homogeneously distributed according to the linear PCE increase. The main functional parameter leading to increased PCE is *J*_sc_, which well correlates with increased dye uptake. In fact, typical increase in *J*_sc_ occurs in DSSCs as a consequence of the increased optical density of the photoanode^29^, which enhances the amount of photogenerated charge, with minor effect on *V*_oc_. We can further observe that the 1@5 sample exhibits the highest dye uptake, compared to 2@4 and 3@3 (Fig. 5a), most probably because the SnO_2_ layer has much higher specific surface, compared to ZnO, according to SEM observations.

As mentioned above, sensitization time can play a relevant role in resulting device performances: while the uptake procedure has been optimized for TiO_2_-based photoanode, attention has to be paid in case of electrodes exploiting other metal oxides as photoelectron carriers^6^. The role of sensitization time *vs* configuration of layered structure can be well understood by considering the trend of quantitative dye uptake *vs* sensitization time for different layer configurations (Fig. 5a) and the subsequent photogenerated current density (shown in Fig. 5b). Samples featuring 1@5 ZnO@SnO_2_ configuration results indeed the most affected by the change in sensitization time: this accounts for an enhanced dye uptake featured by SnO_2_ over the time.

A sensitization procedure lasting 6 h results the best choice for all the investigated electrodes: this is probably the optimal compromise between the need for extremely reduced soaking time required by the ZnO and the longer time needed by the SnO_2_. The main functional parameter affected by dye loading time is the photocurrent (and subsequently the photoconversion efficiency, as clearly shown in Fig. 5d), while *V*_oc_ and FF result almost unchanged with different soaking times.

Electron lifetime (τ_e_) is also affected by the anode architecture: τ_e_ was calculated from the transient photovoltage decay measurements by using the following equation^30^,^31^:
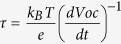


where k_B_ is the Boltzmann constant, T is the absolute temperature, and e is the elementary charge.

**Figure 6:**
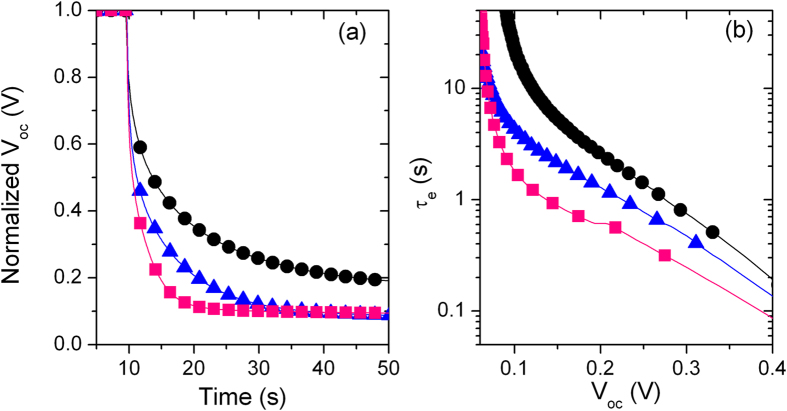
(**a**) Normalized Voc decay and (**b**) electron lifetime in DSSCs with different structure (1@5: black circles; 2@4: blue triangles; 3@3: pink squares).

In the entire *V*_oc_ range, we observe that τ_1@5_ > τ_2@4_ > τ_3@3_ (Fig. 6b). Increased electron lifetime usually calls for reduced charge losses, since the decay of *V*_oc_ under dark reflects the decrease of *V*_oc_ related to internal recombination of the cell in open circuit condition. These results highlight that tailoring the relative number of SnO_2_ and ZnO layers results in electron lifetime modulation.

Electrochemical impedance spectroscopy (EIS) was applied to two layered cells (1@5 and 2@4 ZnO@SnO_2,_ both dye-sensitized for 6h) and to the devices exploiting the single oxides (ZnO and SnO_2_, also dye sensitized for 6 h), aimed at identifying the main electrochemical parameters possibly responsible for the different functional performances.

**Figure 7:**
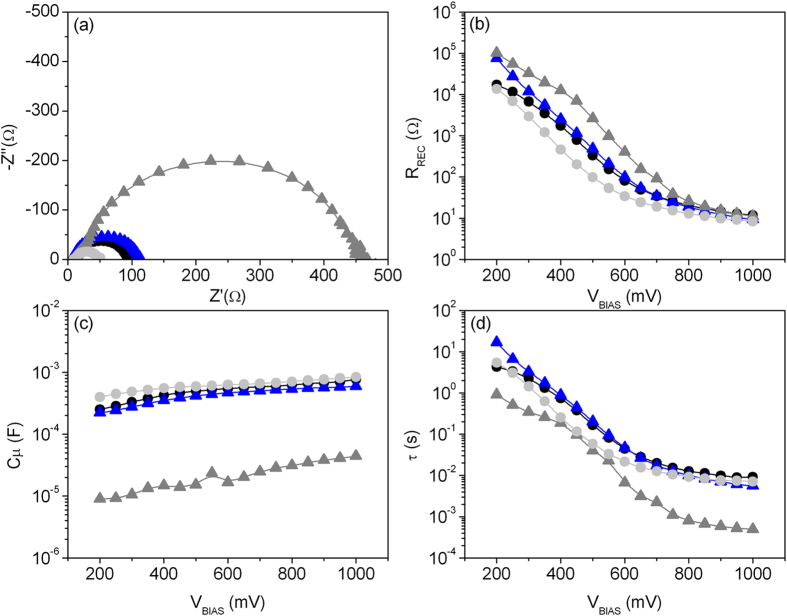
(**a**) Nyquist diagrams of cells at forwarded bias of 600 mV in the dark. (**b**) Recombination resistance between the semiconductors metal oxides and the acceptor species in the electrolyte (**c**) Chemical capacitance Cu and (**d**) electron lifetime t as function of bias. Black line: 1@5 ZnO@SnO_2_; blue line: 2@4 ZnO@SnO_2_; grey line: ZnO; light grey line: SnO_2_.

Main task of EIS analysis was the study of two critical parameters: the chemical capacitance (*C*_μ_) and the recombination resistance (*R*_REC_). *C*_μ_ relates to modified electron density of the semiconductor metal oxide as a function of the Fermi level, whereas *R*_REC_ estimates the recombination between electrons in the photoanode and holes in the electrolyte. These parameters are calculated by using proper equivalent circuit^32^ to fit the experimental data, reported as Nyquist diagram in Fig. 7a.

*V*_*OC*_ is determined by the energy difference between the Fermi level of the metal oxide and the redox potential of the electrolyte, while *J*_SC_ depends on the light harvesting efficiency of dye, its capacity to inject photogenerated charges into the metal oxide conduction band and the charge collection efficiency. *V*_OC_ and *J*_SC_ are intimately correlated^34^: lowering the conduction band edge (*E*_C_) of oxide the *V*_OC_ decrease and *J*_SC_ increase. Raga *et al.*^35^, focused on the relation between the *R*_REC_ with the photovoltage, demonstrating the strong correlation between *R*_REC_ and *V*_*OC*_.

They also highlighted that the differences in *J*_SC_ among different devices are due to the downward shift of *CB* of the oxide (induced by chemical modification of the electrolyte through activating additives), reflecting in the increase of distance between the LUMO in the dye and the *E*_C_, which favors the charge transfer.

In the present study, EIS analysis was carried out within this perspective, i.e. focussed to understand the relation among the above mentioned electrochemical parameters and the device functional behaviour. The observed trend of *R*_REC_ and *C*_μ_ (reported in Fig. 7b,c, respectively) confirms the results of Raga *et al.*^35^. The device exploiting bare SnO_2_ features low *R*_REC_ and high *C*_μ_, with corresponding high *J*_SC_ and low *V*_OC_, as compared with a solar cell whose photoanode is based on pure ZnO, which on the contrary presents high *R*_REC_ and low *C*_μ_ and associated higher *V*_OC_ and lower *J*_SC_.

In the layered bi-oxide photoanode, the functional parameters reported in Table 1 show the effects of the multilayer structure: high *J*_SC_ and high *V*_OC_ are indeed observed when the two oxides are applied. EIS analysis confirms that layered bi-oxide presents good values for both *R*_REC_ and *C*_μ_ (Fig. 7): these architectures simultaneously enable the “good properties” featured by both ZnO and SnO_2_ oxide, which are then allowed to synergistically cooperate with each other and can reach a quite good compromise in terms of functional behaviour.

The enhanced *R*_REC_ observed for layered electrode architectures, as compared with bare SnO_2_, is ascribable to the presence of ZnO, which alone features the best *R*_REC_ among the analyzed devices and, at the same time, exerts a capping effect on the underlying SnO_2_. This result is particularly worth: due to the broad size dispersion of microparticles applied (as highlighted by SEM analysis reported in Fig. 1), coverage of SnO_2_ by ZnO is not conformal, implying that there is, in all devices, SnO_2_ surface exposed to the electrolyte (which favours the exciton recombination at the interface SnO_2_/electrolyte). Despite that, even a partial coverage of SnO_2_ is able to dramatically enhance the *R*_REC_ (and thus the *V*_*OC*_) in bi-oxide photoanodes. This is furthermore confirmed by the trend of *R*_REC_ observed in pure ZnO solar cell, which is the highest in the analysed batch.

As mentioned above, previous studies ascribed the *V*_OC_ enhancement of mixed SnO_2_-ZnO photoanodes (or, generally speaking, SnO_2_-M_x_O_y_ systems) to a more favorable band alignment achieved by engineering a multi-oxide structure, i.e. by a shift of the CB towards energy more favourable for coupling with N719 dye^6^, collaborating also in reducing the back electron recombination at SnO_2_/electrolyte interface.

However, EIS data indicate a different explanation: *V*_OC_ enhancement (beneficial effect due to ZnO) is indeed associated to a decrease in chemical capacitance, as compared with pure SnO_2_ photoanode, as clearly indicated by the trend of *C*_μ_ reported in Fig. 7c. Indeed, we observed an enhancement in *V*_OC_ (1@5 ZnO@SnO_2_ configuration) together with a decrease in *C*_μ_.

EIS analysis further highlights the trend of *R*_REC_ and *C*_μ_ as a function of the number of layers of the two oxides (Fig. 7b,c): increasing the number of ZnO layers reflects in *R*_REC_ increase (which approach the behaviour of bare ZnO) and *C*_μ_ decrease (again moving towards a pure ZnO behavior). On the contrary, by reducing the number of ZnO layers, both *R*_REC_ (decreasing) and *C*_μ_ (increasing) tend to approach a pure SnO_2_ behavior.

These findings are corroborated by a previous electrochemical study, published by Niinobe and coworkers^36^, in which Authors came exactly to the same conclusions reported here, although analyzing a ZnO/SnO_2_ system reproducing that proposed by Tennakone^20^, i.e. a completely different anode architecture. Moreover, Authors even identified an enhancement of back electron recombination correlated with the addition of ZnO to SnO_2_, thus rejecting previous conclusions not based on detailed electrochemical analysis of device behaviour.

The electron lifetime calculated by the equation^30^,^31^:
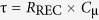
highlights, and further confirms, that the high current obtained in the devices exploiting layered photoanode architectures is not only due to the reduction of recombination rate (indeed pure ZnO-based device presents the worst electron lifetime together with the highest recombination resistance). As shown in Fig. 7d, the trend of electron lifetime featured by 1@5 and 2@4 ZnO@SnO_2_ architectures are more similar to the electron lifetime shown by pure SnO_2_. This further highlights the major role played by the chemical capacitance achieved by the bi-oxide photoanodes: a compromise has to be found between a big reservoir of photogenerated charges and the resistance towards exciton recombination, thus enabling solar energy converting devices featuring high photocurrents together with improved lifetime of photogenerated charges.

**Figure 8:**
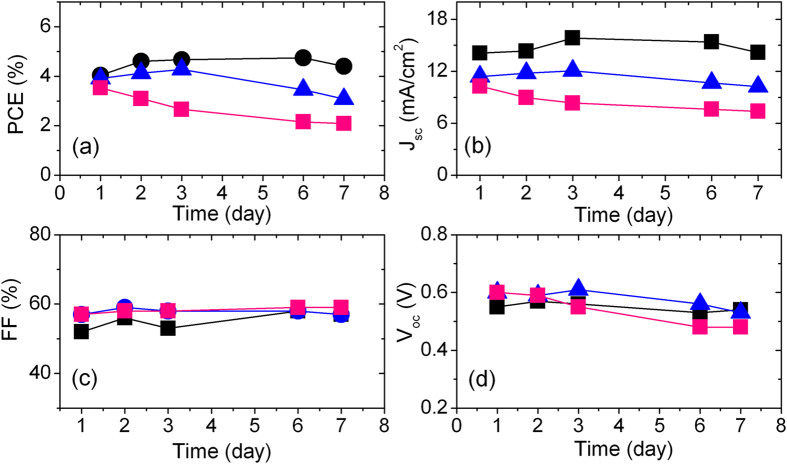
Stability over one week of the devices functional parameters: (a) PCE (%); (b) J_sc_ (mA/cm^2^); (c) FF (%) and (d) V_oc_ (V). Different ZnO@SnO_2_ layers are identified by different markers (1@5: black circles; 2@4: blue triangles; 3@3: pink squares).

DSSCs fabricated by using different configuration of ZnO@SnO_2_ layered architectures, sensitized for 6 h, were subjected to a stability test over seven days (trend of device functional parameters over the time are reported in Fig. 8).

Overall, an acceptable stability was observed for all the devices over one week of observation. In the beginning, for the 1@5 and 2@4 ZnO@SnO_2_ photoanodes configuration, *J*_*sc*_ slightly improved as compared with the current density measured the first day (12% and 6%, respectively) and hence the *PCE* (17% and 10%), whereas for 3@3 configuration both *J*_*sc*_ (13%) and PCE (20%) decreased with respect to *J*_*sc*_ and *PCE* values at the time of device fabrication. So the device with higher SnO_2_ amount features better stability over the time. The device exploiting a 1@5 ZnO@SnO_2_ photoanode configuration is the most stable among the studied batch with minor degradation in *PCE* by only 7% with respect to the best PCE. This is possibly ascribable to enhanced stability towards UV exposure (less oxidative hole is produced in SnO_2_) featured by SnO_2_, which is associated to the corresponding larger band gap of SnO_2_^37^,^38^.

## Conclusions

A very simple approach was applied to fabricate bi-oxide layered architectures to be exploited as photoanodes in dye sensitized solar cells. Layered electrodes delivered better device functional performances as compared with equivalent cells exploiting the single oxides. As compared with pure ZnO and SnO_2_, bi-oxide layered configurations achieved enhancement in both *V*_*OC*_ and *J*_*SC*_, which are main parameters accounting for solar energy conversion. Electrochemical investigation revealed that the enhancement of device functional performances shown by ZnO@SnO_2_-based cells is mainly ascribable to the synergistic effect achieved by the simultaneous exploitation of these two oxides, which collaborate in optimizing the resistance towards the exciton recombination and the capability to accumulate the photogenerated charges at the oxides.

Obtained results indicate that care should be devoted in dye uptake time, while exploiting different oxides than the most commonly applied TiO_2_, for which the dye sensitization procedure has been optimized: modulation of dye loading time is indeed critical to optimize light harvesting and corresponding charge photogeneration.

The present study also highlights the need for accurate electrochemical analyses of excitonic solar cells, aimed at elucidating the actual processes/mechanisms behind the functional performances in the perspective to apply a materials-by-design approach for improving the device capability in converting solar energy.

## Methods

### Chemicals

Tin(IV) chloride (99%), α−terpineol (90%), ammonium hydroxide (28.0–30.0%), methanol (≥99.9%), ethanol (≥99.8%), ethyl cellulose, acetonitrile (99.8%), lithium iodide (99%), Iodine (99.999%), 1,2-dimethyl-3-n-propylimidazolium iodide (≥98.5%) and 4-tert-butylpyridine (96%) were purchased from Sigma-Aldrich Inc. Zinc oxide nanoparticles (10–30 nm, 99.8%) was obtained from SkySpring Nanomaterials, Inc. N719 dye was purchased from Solaronix. Bidistilled water was purchased from Carlo Erba. All chemicals were used as received without any further purification.

### Synthesis of SnO_2_ nanoparticles

In a round bottomed flask 1.2 ml SnCl_4_ were dissolved in 100 ml methanol. After the fumes had disappeared, 4 ml NH_4_OH (30% in water) were added dropwise (addition time about 10 minutes). As soon as NH_4_OH was added, a white flocculate appeared in the reaction mixture. Mixture was let reacting for about 20 minutes, and then the solvent was slowly evaporated at 80 °C in an oven (6–7 h). The raw product was then annealed at 450 °C for 2 h under air atmosphere.

### Preparation of ZnO@SnO_2_ multi-layered photoanodes

SnO_2_ paste was prepared by mixing the appropriate amount of SnO_2_ nanoparticles powder (above prepared SnO_2_, 0.8 g) with ethyl cellulose (0.7 g) and α-terpineol (1.5 ml), in ethanol/water dispersion medium (8/3.5, V/V). The mixture was kept under vigorous stirring overnight to obtain a homogenous paste suitable for tape casting.

Similarly, ZnO paste was prepared by mixing the commercially available ZnO microparticles powder (1 g) with ethyl cellulose (0.5 g) and α-terpineol (1 ml) in a dispersion medium of ethanol/water (5/1, V/V).

The SnO_2_ underlayer was prepared by tape casting the SnO_2_ paste onto FTO glass (sheet resistance 10 Ω/□) and drying it for 10 min at ambient conditions, then at 100 °C for 45 min with a hot plate. ZnO layers were deposited atop the SnO_2_ following the same steps of tape casting as described above. Different thicknesses of the two SnO_2_ and ZnO layers were tested, while keeping constant the overall photoanode thickness (~15–20 μm). Each sample was labeled as X@Y, where X (Y) is the number of ZnO (SnO_2_) cast layers. Each photoanode was composed of 6 layers in total, up to 3 of ZnO. In order to make systematic comparison, single oxide photoanodes (pure SnO_2_ and pure ZnO, samples 0@6 and 6@0, respectively) were also prepared under the same conditions.

All the samples were finally annealed at 450 °C for 30 min under ambient atmosphere. Photoanode thickness was evaluated by stylus profilometry.

All the photoanodes were dye-sensitized by impregnation into 0.5 mM ethanolic solution of commercial Ru-based complex molecular N719 dye for different sensitization times (from 2 h to 10 h) in order to verify the effect of sensitization time. After sensitization, the photoanodes were washed with ethanol to remove excess of unabsorbed dye molecules.

### Device fabrication

DSSCs were fabricated using dye sensitized oxide photoanodes and platinized FTO glass as a counter electrode (5 nm Pt thin film deposited by sputtering) with 60 μm-thick plastic spacers (Surlin from Solaronix). The I_3_^−^/I^−^ redox couple electrolyte was composed of 0.1 M LiI, 0.05 M I_2_, 0.6 M 1, 2-dimethyl-3-n-propylimidazolium iodide, and 0.5 M 4-tert-butylpyridine dissolved in acetonitrile.

### Characterization

UV-Visible measurements were carried out in a T80 spectrophotometer (PG Instruments); quartz cuvettes were used for liquid samples (1 cm optical path). N719 dye was completely detached from the metal oxide photoanodes using a 0.1 M NaOH aqueous solution for dye loading quantification purpose. UV-Visible diffuse reflection (DR) spectra on the powders were measured with a Thermo Fisher Evolution 300 spectrophotometer equipped with a DRA-EV-300 integrating sphere.

Field emission scanning electron microscopy (SEM) analysis was carried out in a LEO1525.

Grazing Incidence X-ray Diffraction pattern was recorded by means of Brüker D8 Advance diffractometer equipped with a Göbel mirror and Cu Kα source (40 kV, 40 mA) at a fixed incidence angle of 1.0°. Crystallite dimension was estimated by Scherrer’s equation.

The current-voltage (I-V) and transient photovoltage decay measurements were carried out by a Keithley 2400 source meter under simulated sunlight irradiation using an ABET 2000 solar simulator at AM 1.5G (100 mW/cm^2^) without masking. The light source was calibrated by using Si solar cell as a reference cell. The active area of the cells was in the range 0.14–0.18 cm^2^. The best performing cells from each configuration were also tested for one week stability and were stored in the dark under ambient conditions during this test.

The electrochemical impedance spectroscopy (EIS) was carried out in dark conditions using a SOLARTRON 1260 A Impedance/Gain-Phase Analyzer, with an AC signal 20 mV in amplitude, in the frequency range between 100 mHz and 300 kHz. External bias in the range 0–1000 mV was applied.

## Additional Information

**How to cite this article**: Milan, R. *et al.* ZnO@SnO_2_ engineered composite photoanodes for dye sensitized solar cells. *Sci. Rep.* **5**, 14523; doi: 10.1038/srep14523 (2015).

## Figures and Tables

**Table 1:** Thickness, functional properties and dye loading of DSSCs exploiting ZnO, SnO_2_ and a hybrid ZnO@SnO_2_ photoanode sensitized for 6 h.

ZnO@SnO_2_(Layer No.)	Thickness(μm)	V_oc_(V)	J_sc_(mA/cm^2^)	FF(%)	PCE(%)	Dye loading × 10^8^(mol/mm^3^)
6@0	20.4	0.67	2.58	60	1.03	−−−
0@6	17.2	0.39	8.00	38	1.20	−−−
3@3	20.4	0.60	10.28	57	3.53	2.04

**Table 2:** Functional parameters, thickness and dye loading quantification of DSSCs based on ZnO@SnO_2_ hybrid photoanodes.

Sensitizationtime (h)	ZnO@SnO_2_(Layer No.)	Thickness(μm)	*V*_oc_(V)	*J*sc(mA/cm^2^)	FF(%)	PCE(%)	Dyeloading × 10^8^(mol/mm^3^)
2	1@5	15.7	0.52	11.4	55	3.26	5.74
	2@4	14.6	0.60	8.9	55	2.95	0.471
	3@3	19.2	0.55	8.5	56	2.64	1.15
4	1@5	20.7	0.56	12.3	57	3.92	5.81
	2@4	18.5	0.56	10.5	56	3.31	2.02
	3@3	20.5	0.56	9.8	56	3.29	2.40
6	1@5	15.0	0.60	14.8	56	4.96	6.07
	2@4	19.8	0.60	11.4	57	3.91	3.74
	3@3	20.4	0.60	10.28	57	3.53	2.04
10	1@5	24.4	0.58	9.0	56	3.60	5.25
	2@4	22.4	0.59	11.8	55	3.89	2.73
	3@3	24.01	0.55	11.1	57	2.83	2.48
